# Biomechanical Signals of Varied Modality and Location Contribute Differently to Recognition of Transient Locomotion

**DOI:** 10.3390/s20185390

**Published:** 2020-09-21

**Authors:** Mahdieh Kazemimoghadam, Nicholas P. Fey

**Affiliations:** 1 Department of Bioengineering, The University of Texas at Dallas, Richardson, TX 75080, USA; mahdieh.kazemimoghadam@utdallas.edu; 2 Department of Mechanical Engineering, The University of Texas at Austin, Austin, TX 78712, USA

**Keywords:** locomotion, biomechanics, task anticipation, mechanical sensing, changes of direction and intent recognition

## Abstract

Intent recognition in lower-limb assistive devices typically relies on neuromechanical sensing of an affected limb acquired through embedded device sensors. It remains unknown whether signals from more widespread sources such as the contralateral leg and torso positively influence intent recognition, and how specific locomotor tasks that place high demands on the neuromuscular system, such as changes of direction, contribute to intent recognition. In this study, we evaluated the performances of signals from varying mechanical modalities (accelerographic, gyroscopic, and joint angles) and locations (the trailing leg, leading leg and torso) during straight walking, changes of direction (cuts), and cuts to stair ascent with varying task anticipation. Biomechanical information from the torso demonstrated poor performance across all conditions. Unilateral (the trailing or leading leg) joint angle data provided the highest accuracy. Surprisingly, neither the fusion of unilateral and torso data nor the combination of multiple signal modalities improved recognition. For these fused modality data, similar trends but with diminished accuracy rates were reported during unanticipated conditions. Finally, for datasets that achieved a relatively accurate (≥90%) recognition of unanticipated tasks, these levels of recognition were achieved after the mid-swing of the trailing/transitioning leg, prior to a subsequent heel strike. These findings suggest that mechanical sensing of the legs and torso for the recognition of straight-line and transient locomotion can be implemented in a relatively flexible manner (i.e., signal modality, and from the leading or trailing legs) and, importantly, suggest that more widespread sensing is not always optimal.

## 1. Introduction

Locomotion is a sequential movement comprising the complex activity of muscular, nervous, and skeletal systems [1]. Neurodegenerative diseases such as stroke and spinal cord injury as well as limb amputation are underlying causes of impaired locomotion, leading to restricted functional independence in the community and reduced quality of life [2,3,4]. Advanced wearable assistive devices (i.e., lower-limb prostheses, orthoses/exoskeletons) have been impressively developed over the last decade to restore or enhance motor function of the affected limbs [5,6,7]. To enable an effective human–device interaction, intent recognition strategies have been successfully developed to infer user intention for the control system and to actuate the device accordingly [7,8,9]. The accurate and timely prediction of user locomotion mode can be necessary to maximize the benefits of these devices and to ensure user safety. However, the performance of the recognition system is highly dependent on the sensor information that is used as an input for the device control. In nearly all commercially available assistive devices and those used in research settings, mechanical sensors are typically embedded in the device. For instance, in the Vanderbilt powered prosthesis, data recorded through built-in potentiometers, encoders, six-axis inertial measurement units (IMU), and load cells on the shank were used to control the prosthesis according to the wearer’s intentions to move [10]. In the Hybrid Assistive Limb, the hip and knee joint angles were used as input commands to control the device [11]. Commercialized devices such as C-Leg knee prosthesis and C-Brace orthosis are also controlled using signals from embedded mechanical sensors [12,13]. In the users of these devices, the unaffected or less affected portions of the body (e.g., contralateral leg, upper body) remain functional and could serve as beneficial sources of sensing information. However, little information exists on potentially using biomechanical data from the intact limb for detecting an individual’s intended motion, and it remains unknown whether this information would be dependable for intent recognition frameworks. The answer to this question could provide insight into determining reliable, simple, and flexible sources of information for the intuitive operation of assistive devices. 

Complementary limb motion estimation (CLME) is the idea that an assistive device motion can be estimated based on the motion of the intact leg using the concept of inter-joint coupling. CLME was first implemented in rehabilitation robots to generate reference trajectories [14], then to control prosthetic legs [15] where missing limb trajectories were determined by measuring contralateral leg joint angles and angular velocities. However, this method has only been implemented for joint actuation and position control, and there has not been much information on whether signals from the intact limb(s) could provide timely and accurate recognition of upcoming locomotion mode or lower-limb activities. Furthermore, intent recognition of human locomotion has been primarily limited to the detection of basic steady-state ambulation such as straight level-ground walking, ramp and stair ascent/descent, and transitions between level and uneven terrain (e.g., ramp/stair to level ground and vice versa) [7,8,9]. 

The recognition of non-steady (i.e., transient) changes of direction, such as turns and cuts, occurring frequently in community ambulation and many recreational activities has not been demonstrated. For example, sidestep and crossover cuts are the most frequently performed cutting styles and defined by when the torso moves toward the opposite or same direction as the stance leg, respectively. During cuts, excessive stress is placed on the joints to coordinate body segments and muscles and maintain stability [16]. Hence, compared to the tasks performed in primarily one plane of progression, the risks of fall and injury are higher during cuts due to relatively high levels of neuromotor demand [17]. A more complex, combined cut and stair ascent transition requires both horizontal and vertical alterations placing even higher demands on the neuromuscular control system. The control of dynamic stability and balance was shown to further deteriorate when such maneuvers were performed under unanticipated conditions [18,19]. Anticipatory neuromotor control is dependent on sensory inputs and is significantly influenced by an individual’s knowledge of upcoming motion, and their ability to integrate sensory information. During anticipated locomotor tasks, individuals perform preparatory adaptations such as altering their step size and joint moments [20,21]. In contrast, in unanticipated tasks, studies suggest reflexes are required to prevent falls, such as following an unexpected perturbation [16,20]. The biomechanical challenges of these tasks and other transient lower-limb tasks, especially when performed by individuals with sensory/cognitive impairments, further emphasize the need to identify informative yet flexible data sources to seed controllers of lower-limb assistive technologies. 

In this study, we conducted an offline analysis to comprehensively compare the performance of altering mechanical sensing modality (accelerographic, gyroscopic, joint angles, and their combinations) and location (the trailing leg, leading leg, torso and their fusion) to recognize multiple forms of straight-line and transient locomotion that are performed under varying levels of task anticipation. Specifically, we continuously classified five categories of locomotion, including straight walking, 45° sidestep and crossover cuts, and cuts to stair ascent transitions performed under anticipated and unanticipated states. We tested the hypothesis that signal fusion from different modality and body location would lead to improved accuracy compared to signals from isolated locations and/or modality. We further hypothesized that the performance of a given signal would diminish when unanticipated tasks were performed.

## 2. Materials and Methods

### 2.1. Subjects and Data Collection

**Figure 1:**
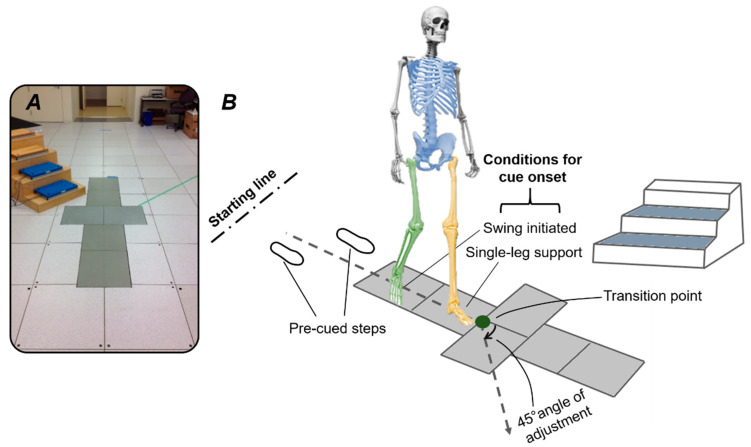
Experimental setup for the locomotor tasks. (**A**) Lab setup for straight walking, sidestep and crossover cuts, and cuts to stair ascent performed under varying task anticipation. (**B**) The transition point and path for cut transitions were shown on the walkway. Each trial was initiated with the first trailing leg heel strike. Subjects entered a locomotor transition at the first trailing leg toe off, and transition ended with the second trailing leg heel strike. Unanticipated trials were recorded when a randomized cue was given half a step prior to the marked transition point on the terrain. Different signal locations, including the trailing leg, leading leg, and trunk-pelvis were indicated with green, yellow and blue, respectively.

Non-disabled, healthy subjects (4 females, 1 male, average age: 27.7 ± 3.8 years, mass: 52.6 ± 2.8 kg, height: 1.68 ± 0.06 m) participated in the study. Subjects did not have a history of neuromuscular disorders or injuries. The experimental procedures used for this study were reviewed and approved by an Institutional Review Board, and informed consents were obtained from the subjects before participating in the experiments. The tasks selected for this study included straight walking at comfortable speed (W), crossover (CO) and sidestep (SS) cuts, crossover to stair ascent (COS), and sidestep to stair ascent (SSS). All locomotor transitions were performed under anticipated (A) and unanticipated (UA) conditions. The lab setup consisted of a level straight walkway, a level 45° walkway to the right for sidestep and crossover cuts, and a portable staircase at 45° to the left for the task with stair-ascent transitions (Figure 1A). Each subject completed five straight-walking trials, followed by five trials of each anticipated cut and cut-to-stairs transition. Similar to anticipated conditions, unanticipated conditions were comprised of five trials of unanticipated walking and five trials for each locomotor transition. Unanticipated trials were recorded when a randomized auditory cue for “walk”, “cut” and “stair” was given at the beginning of the trailing leg swing phase and half a step prior to the marked transition point on the terrain (Figure 1). The position of each leg when the cue was given was used to define the trailing and leading legs. Each trial was initiated with the first trailing leg heel strike (THS1); subjects executed cut transitions with the first trailing leg toe off (TTO1) and ended with the second trailing leg heel strike (THS2). In cut-to-stairs transitions, stair ascent was initiated with the second leading leg toe off and ended with the third trailing leg heel strike. Forty-two reflective markers were placed on the subject’s bilateral foot, shank, thigh, as well as trunk and pelvis. Gait kinematics in three dimensions were captured using a ten-camera motion capture system (Motion Lab Systems, Baton Rouge, LA, USA) operating at 120 Hz. 

### 2.2. Signal Processing and Recognition Performance

Kinematic signals, including joint angles and segments linear acceleration and angular velocity, were recorded from the trailing leg, leading leg and torso. Data were processed using Visual3D (C-Motion, Germantown, MD, USA) and exported to MATLAB (Mathworks, Natick, MA, USA) for further analysis. An offline study was conducted to determine the benefit of different input signal modalities and locations for predicting intent. Signals were divided into seven modality groups, including linear acceleration (LA), angular velocity (AV), joint angle (ANG), combinations of two kinematic signals (LA+AV, LA+ANG, AV+ANG), and combination of all three signals (ALL). Signals were also divided into four location groups (Figure 1B) comprising the trailing leg, leading leg, trunk–pelvis, and all combined (fusion). The trailing/leading leg signals included linear acceleration and angular velocity of the foot, shank, and thigh, as well as ankle and knee angles (sagittal plane only). The trunk–pelvis signals were comprised of linear acceleration and angular velocity of the trunk and pelvis, and trunk angle (sagittal plane only).

Linear Discriminant analysis classifier (LDA) was used to continuously classify the previously introduced locomotion modes. LDA has been the benchmark for intent recognition in lower-limb prostheses control [9,22] and has shown to be a good compromise between classification accuracy and computational efficiency. All signals were divided into sliding and overlapping analysis windows. Windows of 100–600 ms with a 25 ms overlap were tested to find the optimal window size for this study. Our statistical analysis did not report significant main effects related to window size for the selected tasks (p = 0.83–0.92). Nonetheless, a greater window length would increase the time delays in recognizing the locomotion mode, which should be taken into account in intent recognition frameworks, where such delays could compromise real-time analysis. Smaller window sizes are a more suitable option for quick movements such as the tasks presented in this study. Therefore, the nominal window of 200 ms was used in this study. Subsequently, six time domain features, including minimum, maximum, mean, standard deviation and first and last sample of the window were extracted from each individual window. These features are computationally inexpensive and have functioned relatively well in real-time control of lower-limb assistive device [7,10,22,23]. For classifying anticipated locomotor tasks, the classifier was trained with only anticipated data. For unanticipated tasks, it was trained with all anticipated and unanticipated trials [24]. Leave-one-out cross validation was applied to each subject’s data. At each validation step, one trial of a given transition was excluded from the training set and was used to test the classifier. This was repeated to test all trials of each transition. The results were then averaged across the subjects. The influence of signal location and modality on classification performance was studied by analyzing the classification accuracy as well as error patterns over time. To calculate classification accuracy, the number of correctly identified windows starting at TTO1 was divided by the total number of windows during TTO1 and the end of the locomotion, and it was reported as a percentage (1). TTO1 is when the subjects entered a locomotor transition.

(1) $$Accuracy=\frac{number\;of\;correctly\;classified\;windows\;begining\;toe\;off}{total\;number\;of\;windows\;}\times 100$$

Error patterns versus time were plotted by allocating either zero (correctly classified) or one hundred (incorrectly classified) to every single analysis window starting at THS1. The corresponding windows of the trials were then averaged.

The Shapiro-Wilk test was used to check the normality assumption of these data. Then, two-way ANOVA was performed with the accuracy rate as the response variable, and signal modality and location as fixed factors. If the ANOVA revealed a significant result, post-hoc testing using pairwise comparison with Bonferroni correction was performed. The alpha level was set to 0.05 for all tests. Cohen’s effect size (ES) was calculated to assess the magnitude of the difference [25]. Generally, the larger the effect size, the greater the difference between the two groups is. A value of at least 0.8 is considered to be a large (positive) effect, 0.5 is medium, and 0.2 is small. Furthermore, power analyses were performed for statistically significant combinations of signal modalities and locations. At an alpha of 0.05, the null hypothesis (i.e., there is no true difference) was rejected when statistical power exceeded 80%. 

## 3. Results

### 3.1. Classification of Anticipated Locomotor Tasks

**Figure 2:**
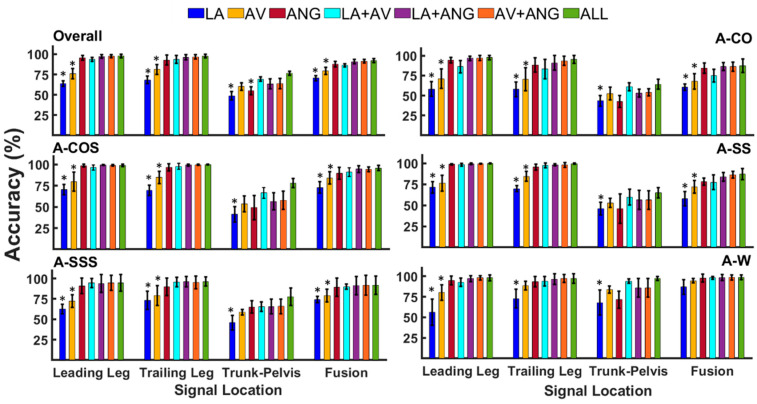
Classification accuracy of anticipated straight walking (A-W), crossover and sidestep cuts (A-CO, A-SS), cuts to stair ascent (A-COS, A-SSS), as well as overall accuracy across transitions using signals of varying modalities (LA, AV, ANG, LA+AV, LA+ANG, AV+ANG, ALL) and locations (the leading leg, trailing leg, trunk–pelvis, and fusion across locations). Asterisks denote modalities that were statistically different from signal combinations (alpha = 0.05).

There was a significant interaction effect between modality and location (p < 0.001). During cuts and cut-to-stairs transitions, the maximum classification accuracy of a given signal modality was achieved when the data were captured from the trailing or leading leg (Figure 2, Table 1). Signals from the leading and trailing legs resulted in relatively identical accuracy rates (p = 0.25–0.95, ES = 0.2–0.7). Fusion of unilateral (the trailing/leading leg) and trunk–pelvis data appeared to provide either lower (p = 0–0.03, ES = 1.1–2.8) or statistically similar (p = 0.17–0.96, ES = 0.3–0.5) outcomes relative to using only unilateral signals. Trunk–pelvis signals showed inferior performance to unilateral signals (p < 0.001, ES = 1.6–7.8). Only in anticipated straight walking (A-W) did signals captured from the trunk–pelvis provide similar outcomes to those of unilateral information (p = 0.06–0.8, ES = 0–0.2).

Within the trailing and leading legs, using linear acceleration (LA) and angular velocity (AV) data showed the lowest performance in all locomotor tasks (Figure 2, Table 1). All combinations of two modalities (LA+AV, LA+ANG, AV+ANG) as well as combination of three modalities (ALL) outperformed LA and AV (p < 0.001, ES = 0.9–5.8). LA and AV from the leading leg provided overall accuracies of 63.8% and 75.5%, respectively, and accuracies of 68.4% and 81.1% were reported when the signals were captured from the trailing leg. Using unilateral joint angles (ANG), significantly improved the overall accuracy to 95.5% and 92.7% for the leading and trailing leg signals, respectively. A maximum overall accuracy of 97% was achieved using combination of all three signal modalities (ALL). However, statistical analysis did not reveal any significant differences when ANG, ALL, and any combinations of two modalities (LA+AV, LA+ANG, AV+ANG) were compared (p = 0.33–0.94, ES = 0–0.7).

**Figure 3:**
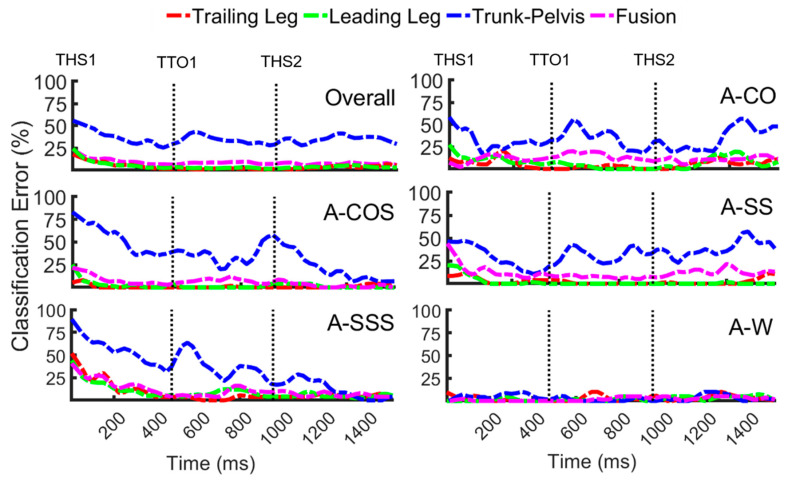
Classification error over time for anticipated locomotor transitions (A-CO, A-COS, A-SS, A-SSS, and A-W) as well as overall error across transitions using biomechanical signals from the trailing leg, leading leg, trunk-pelvis and their fusion. Each trial was initiated with the first trailing leg heel strike (THS1). Subjects entered a locomotor transition at (TTO1), and transition ended with the second trailing leg heel strike (THS2).

To compare the error rates of signals from different locations over time, ALL data captured from the leading leg, trailing leg, trunk–pelvis, and their fusion were plotted versus time (Figure 3). Decreasing error trends were observed as the locomotion was initiated (i.e., THS1) using the leading leg, trailing leg, and fusion signals. In cuts and cut-to-stairs modes, the error trends reached their minimum and leveled off approximately 300–400 ms prior to entering a locomotor transition (i.e., TTO1). As the transition continued, overall error values remained between 1 and 5% for the unilateral signals and 5–10% for the signal fusion. Highly overlapping trends were obtained, especially using the trailing and leading leg data. Trunk-pelvis showed the highest levels of error and fluctuation during all anticipated tasks, except in A-W, where errors remained between 0 and 3% over time, and all error patterns appeared to be highly overlapping.

### 3.2. Classification of Unanticipated Locomotor Tasks

**Figure 4:**
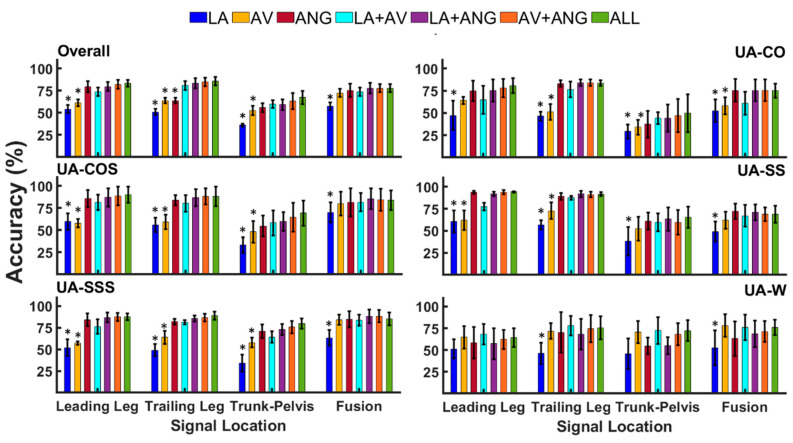
Classification accuracy of unanticipated straight walking (UA-W), crossover and sidestep cuts (UA-CO, UA-SS), cuts to stair ascent (UA-COS, UA-SSS), as well as overall accuracy across transitions using signals of varying modalities (LA, AV, ANG, LA+AV, LA+ANG, AV+ANG, ALL) and locations (the leading leg, trailing leg, trunk–pelvis, and fusion across locations). Asterisks denote modalities that were statistically different from signal combinations (alpha = 0.05).

There was a significant interaction effect between modality and location (p < 0.001) in unanticipated transitions. Similar to anticipated tasks, using signals from the trailing/leading leg (unilateral) led to higher accuracies than trunk–pelvis (p < 0.001, ES = 1.6–7.8) (Figure 4, Table 2). Signal fusion provided either statistically similar (p = 0.26–0.96, ES = 0.3–0.5) or lower accuracy rates relative to unilateral signals. The trailing and leading leg information led to relatively identical outcomes (p = 0.58–0.95, ES = 0.2–0.7). In addition, the trunk–pelvis data appeared to have the most inferior performance in all locomotion modes except in UA-W, where they provided statistically similar outcomes to those of unilateral and fusion data (p = 0.26–0.94, ES = 0–0.4).

Within the trailing/leading leg, LA and AV provided the most inferior performance in different locomotor tasks (p < 0.001, ES = 0.7–8.8). LA and AV showed lower accuracy relative to all combinations of two (LA+AV, LA+ANG, AV+ANG) and three (ALL) modalities (p < 0.001, ES = 0.7–8.8). Only in UA-W unilateral LA and AV showed comparable outcomes to other modalities (p = 0.05–0.94, ES = 0–0.6). Statistically similar performances of ANG, combinations of two signal modalities (LA+AV, LA+ANG, AV+ANG) and ALL were observed in all the tasks (p=0.05–0.96, ES= 0–0.7). Finally, lower accuracy rates were reported for unanticipated tasks relative to anticipated. For instance, using ALL data from the leading and trailing leg led to overall accuracies of 83.3% and 85.5%, respectively, which were significantly lower compared to those of anticipated modes (97.8%, 97.5%). Similar outcomes were observed when the accuracies of other signal modalities were compared across anticipated and unanticipated states. 

**Figure 5:**
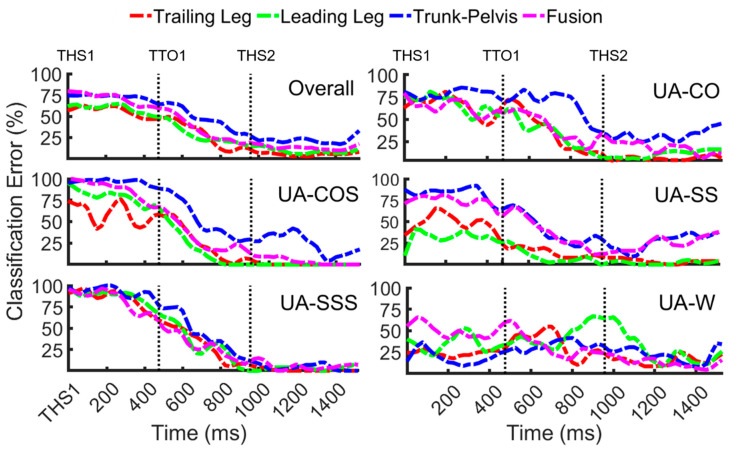
Classification error over time for unanticipated locomotor transitions (UA-CO, UA-COS, UA-SS, UA-SSS, and UA-W) as well as overall error across transitions using biomechanical signals from the trailing leg, leading leg, trunk-pelvis and their fusion. Each trial was initiated with the first trailing leg heel strike (THS1). Subjects entered a locomotor transition at (TTO1), and transition ended with the second trailing leg heel strike (THS2).

Error patterns of unanticipated modes versus time remarkably differed from those of anticipated tasks (Figure 5). Error levels were relatively high at the beginning of the locomotion and before TTO1 (25–95%). Downward trends were observed only after entering a locomotor transition (TTO1). Overall error patterns reached their minimum around 200 ms prior to the subsequent heel strike (THS2) and remained in the range of 2–14%, 10–20% and 17–30% for unilateral, fusion, and trunk–pelvis data, respectively. Nonetheless, different patterns were observed during UA-W; the trends appeared to be highly fluctuating across time in this mode, implying the fact that UA-W could be the hardest task to classify. 

## 4. Discussion

Determining the modality and location of mechanical sensing, which provide strong discrimination of straight-line and transient locomotion, performed in anticipated and unanticipated states is needed to guide the control of assistive technologies. Sensing that is flexible and reliable is important, in particular, during challenging forms of locomotion that increase the biomechanical demand and pose significant fall risks. The purpose of this study was to comprehensively compare the performance of altering mechanical sensing modality (accelerographic, gyroscopic, joint angles and their combinations) and location (the trailing leg, leading leg, torso and their fusion) to recognize multiple forms of straight-line and transient locomotion that are performed under varying levels of task anticipation. 

Our first hypothesis regarding improved outcomes using the fusion of signals across different locations/modalities was not supported. The fusion of the unilateral (the trailing/leading leg) and torso data resulted in either similar or lower accuracy compared to only unilateral signals. Within unilateral information, even though signal combinations outperformed accelerographic and gyroscopic data alone in most cases, they appeared to provide comparable accuracy to joint angles. The results suggest that when use of a single-modality data is desired, joint angles would be a better choice than gyroscopic and accelerographic information for discriminating between the selected maneuvers. The combination of gyroscopic and accelerographic information provided statistically similar accuracy to the combination of each of these signals with joint angles. Chung et al. [26] confirmed similar outcomes in a set of daily activities, where different combinations of two sensor modalities showed comparable task recognition accuracy. These findings could provide flexibility in selecting appropriate combinations of input signals depending on hardware availability and/or patient convenience in assistive device applications. Furthermore, the performance of a single-modality signal could differ depending on the characteristics of the activity. Thus, using such combinations could help in compensating the imprecision associated with each modality, leading to improved generalizability of the outcome to varying target tasks [27]. 

According to these findings, neither the combination of all three modalities nor the fusion of torso and lower-body data appeared to be advantageous. This is consistent with the results of [28], where adding extraneous sensors was shown to deteriorate task recognition. These findings may suggest that, in assistive device control, recognition accuracy may not increase as more sensors are added. We expect these trends to be due to overfitting which occurs when the number of input signals increases. In such scenarios, classification models learn the details in the training data which negatively impacts the model’s ability to generalize to new data [29]. From a practical perspective, optimizing the number of input signals and identifying sources capturing less important or redundant information would avoid attaching additional sensors on the subject, reduce system complexity, make the design less encumbering for the wearer, and improve the efficiency of the design for its eventual clinical use. Furthermore, the trailing and leading leg data appeared to have a similar performance, suggesting that, regardless of whether an assistive device is on the leading or training leg, contralateral signals could be sufficient to provide an accurate recognition. Hu et al. [28] reported similar outcomes using wearable sensors where ipsilateral and contralateral leg sensor sets demonstrated comparable performances. Previous studies have demonstrated that using upper-body data (e.g., waist-mounted or pelvic sensor) would reduce the likelihood of data loss without restricting the user’s mobility or their daily activities [30,31]. However, according to our findings, signals from the trunk–pelvis had poor task generalization. This could be due to the significantly smaller degrees of torso movement and decreased ranges of motion compared to lower-limb segments (e.g., ankle, knee) [32]. A small amount of trunk/pelvis movement presents low inter-class variability, posing a more difficult classification problem [33]. Hence, for cuts and cut-to-stairs transitions, recording biomechanical data from the contralateral side (the trailing/leading leg) could be a tradeoff for better accuracy, which may be more desirable for some subjects. 

Our second hypothesis was supported. Regardless of the types of input signals, improved performance (~15–25%) was observed in anticipated relative to unanticipated tasks (Table 1 vs. Table 2). Anticipatory biomechanics modifications to the upcoming locomotor task occur as early as three strides in advance of a transition [20,21]. Kinematic adaptations such as changes in step width during the preparatory phase were shown to significantly improve stability during anticipated maneuvers [34,35]. In contrast, in unanticipated locomotor tasks, a lack of preparatory adaptations does not allow individuals to plan and organize their gait modifications in the most optimal way. A reduced stability margin in such maneuvers has been shown to be associated with substantial kinematic variability. This leads to high intra-class variation, posing a difficult classification problem [33], which could justify reduced accuracy rates in unanticipated locomotion. Early biomechanical adaptations could also explain relatively low error rates even prior to entering a locomotor transition (i.e., trailing leg toe off, TTO1) in anticipated tasks (Figure 3). From a practical standpoint, early detection of the locomotion mode would provide adequate time for the assistive device control system to adjust its parameters in a smooth manner to accommodate the upcoming heel strike. However, in unanticipated tasks, a lack of preparatory locomotor adaptations prior to executing transitions resulted in relatively high error rates (≥25%) until the mid-swing of the transitioning leg. Nonetheless, if the user wore the device on the transitioning leg, we expect there could be sufficient time to switch the device modes during the subsequent second half of swing, and prepare for weight bearing, which is encouraging. 

We acknowledge some specific limitations of this study. For instance, in order to detect unanticipated tasks, substantial amount of unanticipated information (i.e., five bouts) were included in the training data. We expect that accuracy rates for unanticipated locomotion modes would diminish using training data with reduced number of target task repetitions [24]. Thus, the potential impact of training data on the outcomes should be taken into consideration when selecting optimal signal sources. In this study biomechanical data were extracted using a camera-based motion capture system, and we have not explored these relationships using physical sensors. Nonetheless, motion capture data are not noise-free and suffer from many of the same issues as mechanical sensors such as high-frequency motion artifacts and determining consistent and accurate sensor placement. Moreover, previous work has shown that kinematics measurements from body-worn sensors are comparable to those captured using motion capture [36]. Input signal locations providing the optimal outcomes were also reported to be the same in both methods [37]. Thus, we expect that the trends observed in this study will have many important implications. However, issues associated with the actual wearable sensors such as difficulties with aligning goniometers and their crosstalk [38], and inherent bias and drift errors in actual IMUs and gyroscopes [39], may affect system performance, and should be taken into consideration in future studies. Finally, this study included data from able-bodied individuals. Future studies investigating sensor data from subjects with mobility impairments (e.g., lower-limb amputees) would shed further light on these relationships between sensing modality, sensing location and locomotor task. 

## 5. Conclusions

For both anticipated and unanticipated conditions, joint angle information showed a better performance than gyroscopic and accelerographic information (20–60% higher accuracy) for discriminating the widely varying locomotor tasks. While only joint angle data from the trailing or leading leg appeared to be sufficient for an accurate recognition (accuracy = 80–98.7%), biomechanical information from the torso demonstrated poor task generalization. Lastly, signal fusion across multiple locations and modalities did not lead to improved task discrimination. These findings provide fundamental insights that are relevant for multiple applications of assistive and activity-monitoring technologies.

## Figures and Tables

**Table 1:** Classification accuracy of anticipated locomotion modes (A-CO, A-COS, A-SS, A-SSS, A-W), as well as overall accuracy across transitions using signals of varying modalities and locations. Values represent the mean (standard deviation).

	Signal Location	LA	AV	ANG	LA+AV	LA+ANG	AV+ANG	ALL
**Overall**	Leading Leg	63.8 (3.5)	75.9 (6.3)	95.5 (2.8)	93.6 (2.4)	97.2 (2.3)	97.6 (2.05)	97.8 (2.3)
	Trailing Leg	68.4 (4.6)	81.1 (6.1)	92.7 (6.1)	93.5 (4.7)	96.2 (3.1)	96.7 (2.8)	97.5 (2.2)
	Trunk-Pelvis	48.8 (5)	60.2 (4.5)	54.8 (4.9)	69.4 (2.5)	63.4 (6.1)	63.9 (6.2)	76.4 (2.5)
	Fusion	70.4 (3.2)	79.4 (4.4)	87.8 (3.4)	86.3 (1.9)	90.8 (2.6)	91.3 (2.3)	92 (2.4)
**A-CO**	Leading Leg	58.2 (9)	71.1 (11.9)	94.5 (3.5)	86.2 (7.6)	96.7 (2.6)	97 (3.2)	97.5 (2.8)
	Trailing Leg	57.7 (9)	70.5 (14.6)	88.3 (8.8)	83.1 (12.1)	91 (9)	93.5 (5.6)	95.2 (5)
	Trunk-Pelvis	43.3 (6.7)	52.3 (8)	42.4 (7.5)	61.1 (5)	53 (5)	54.1 (4.5)	64 (6.2)
	Fusion	60.4 (4)	67.7 (9.5)	84.4 (6.3)	74.8 (7.8)	86.2 (4.8)	86.2 (5.6)	87.2 (8.4)
**A-COS**	Leading Leg	70.5 (6.2)	79.8 (11.2)	98.7 (1.8)	96.5 (3)	99.3 (0.4)	98.8 (0.9)	99.1 (1.3)
	Trailing Leg	69.6 (6)	84.9 (7.2)	96.5 (4.1)	97.7 (3.5)	99.2 (1)	99.6 (0.7)	99.7 (0.4)
	Trunk-Pelvis	41.3 (9)	53.7 (9.3)	49.1 (14)	66.6 (6.2)	56.5 (10)	57.8 (10.5)	78.1 (5.4)
	Fusion	72.9 (6.7)	84.1 (7.1)	89.8 (7)	91.1 (5.2)	94.6 (4)	94.3 (2.8)	95.8 (3.1)
**A-SS**	Leading Leg	71.4 (6.8)	76.4 (9.4)	98.7 (0.7)	98.3 (1.6)	99.4 (0.8)	99.4 (0.4)	99.7 (0.5)
	Trailing Leg	69.8 (3.4)	84.4 (5.9)	95.5 (3.6)	97.4 (2.7)	98.1 (1.2)	97.9 (2.6)	99.4 (0.7)
	Trunk-Pelvis	45.9 (7.4)	52.7 (5.4)	45.9 (17)	59.7 (9.5)	56.5 (11)	56.2 (11)	64.9 (6.2)
	Fusion	57.7 (8.5)	72.04 (7.5)	78 (4.2)	77.3 (8.6)	83.6 (5.1)	86.2 (4.1)	86.9 (6.5)
**A-SSS**	Leading Leg	62.5 (5.6)	72.1 (7.7)	91 (9.6)	94.4 (5.3)	93.9 (10.9)	94.7 (9)	94.6 (10)
	Trailing Leg	73.16(11)	79.16 (12)	89.8 (10)	95.7 (5.7)	96.2 (6.1)	95.2 (7.8)	96.2 (5.6)
	Trunk-Pelvis	45.7 (9.1)	58.6 (3.5)	65.2 (7.7)	65.5 (5.8)	65.5 (9)	65.7 (9)	77.6 (10)
	Fusion	74 (3.9)	78.9 (7.8)	89.2 (11)	90 (3)	91.3 (11)	91.5 (12)	91.7 (11)
**A-W**	Leading Leg	56.2 (16)	80.2 (9.4)	94.8 (5.3)	92.6 (4.8)	97 (3.6)	98 (2.7)	98 (3.4)
	Trailing Leg	72.6 (11)	88.3 (5.2)	93.2 (6.2)	93.7 (5.7)	96.4 (6.4)	97.3 (4.7)	97.1 (5.7)
	Trunk-Pelvis	68 (15)	83.5 (4.2)	71.4 (10)	94 (2.7)	85.7 (11)	85.7 (11)	97.3 (2.3)
	Fusion	86.8 (9)	94.5 (2.6)	97.4 (4.8)	98.15 (1.6)	98.4 (3.5)	98.5 (3.2)	98.5 (3)

**Table 2:** Classification accuracy of unanticipated locomotion modes (UA-CO, UA-COS, UA-SS, UA-SSS, UA-W), as well as overall accuracy across transitions using signals of varying modalities and locations. Values represent the mean (standard deviation).

	Signal Location	LA	AV	ANG	LA+AV	LA+ANG	AV+ANG	ALL
**Overall**	Leading Leg	54.1 (4.5)	61.2 (3.7)	79.4 (6.1)	73.7 (4.7)	79.6 (5.1)	81.9 (5.1)	83.3 (3.6)
	Trailing Leg	50.8 (3.5)	63.8 (3.1)	63.8 (3.1)	80.7 (4.8)	83.3 (5.7)	84.6 (4.7)	85.5 (4.7)
	Trunk-Pelvis	36.1 (1.6)	52.6 (5.2)	55.8 (5.1)	59.8 (4.5)	59.1 (6)	63 (9.1)	67.2 (7.2)
	Fusion	57.4 (4.3)	72.4 (4.6)	75.3 (7.4)	73.8 (4.8)	77.7 (5.8)	77.6 (4.6)	77.7 (4.4)
**UA-CO**	Leading Leg	47.2 (16)	64.1 (3.8)	75 (11.5)	64.9 (15.7)	75.2 (12.3)	78 (10.1)	80.7 (8.2)
	Trailing Leg	46.4 (5)	51.4 (8.8)	83 (3.5)	76.5 (8.7)	84 (3.5)	84.1 (3.6)	83.6 (3.1)
	Trunk-Pelvis	29.1 (7.8)	33.9 (8.3)	37.4 (15)	44.3 (6.8)	44.3 (15)	47 (18)	49.8 (21)
	Fusion	52.4 (12)	57.9 (9.5)	75.4 (12)	60.9 (13)	75.5 (12)	75.4 (12)	75.2 (7)
**UA-COS**	Leading Leg	59.6 (9.2)	57.8 (4.6)	85.8 (9.5)	81.3 (8.7)	87 (10.3)	88.6 (10.6)	89.8 (9.1)
	Trailing Leg	55.9 (8)	59.2 (8.2)	83.7 (5.8)	80.4 (9.3)	86.7 (9.5)	88.1 (8.9)	88.1 (11)
	Trunk-Pelvis	33.3 (9)	48.1 (12)	54.6 (11)	58.3 (14)	59.7 (10)	64.5 (16)	69.2 (14)
	Fusion	70 (11)	79.9 (13)	81.3 (15)	81.6 (10)	85.3 (11)	84.4 (12)	83.7 (11)
**UA-SS**	Leading Leg	60.5 (12)	62.16 (11)	93.6 (1.7)	77.5 (4.3)	92 (2.3)	93.6 (2.3)	94.1 (0.8)
	Trailing Leg	56.7 (5.3)	72.7 (9.3)	89.2 (3.8)	87.4 (2)	91.7 (3.7)	91 (3.1)	91.5 (2)
	Trunk-Pelvis	38.2 (16)	52.2 (13)	61 (9.5)	59.7 (10)	63.2 (13)	59.5 (13)	65.3 (11)
	Fusion	49.1 (11)	61.9 (9.5)	72.2 (8.6)	66.9 (12)	71 (8.8)	68.8 (7.7)	68.6 (9.5)
**UA-SSS**	Leading Leg	51.9 (9.7)	57.2 (2)	84.1 (7.5)	76.3 (8.1)	86.9 (5.5)	87.4 (4.4)	87.7 (4)
	Trailing Leg	49 (7)	63.8 (7.7)	82.2 (3.2)	81.3 (2.5)	85.6 (3.4)	86.5 (4.4)	89 (4.6)
	Trunk-Pelvis	34.1 (9.9)	57.8 (5.6)	71.1 (7.9)	64.2 (6.6)	73 (6.3)	75.6 (6.9)	79.8 (5.9)
	Fusion	63.1 (9)	84.1 (5.8)	84.7 (9.3)	83.58 (6.3)	88.15 (7.9)	88.2 (7.1)	85.2 (7.5)
**UA-W**	Leading Leg	51.2 (10)	64.5 (12)	58.4 (17)	68.3 (11)	57.3 (17)	62.1 (11)	64.2 (10)
	Trailing Leg	46.2 (12)	71.7 (9)	70.3 (23)	77.8 (11)	68.2 (17)	74.5 (15)	75.3 (13)
	Trunk-Pelvis	45.7 (17)	70.8 (12)	54.7 (9.7)	72.4 (15.5)	55.1 (9.7)	68.1(12.8)	72.2 (11.8)
	Fusion	52.5 (20)	78 (13)	63.1 (20)	76 (15)	68.7 (15)	71.2 (12)	75.8 (9)
